# Kawasaki Disease

**DOI:** 10.3389/fped.2018.00198

**Published:** 2018-07-10

**Authors:** Christian M. Hedrich, Anja Schnabel, Toni Hospach

**Affiliations:** ^1^Department of Women's and Children's Health, Institute of Translational Medicine, University of Liverpool, Liverpool, United Kingdom; ^2^Department of Paediatric Rheumatology, Alder Hey Children's NHS Foundation Trust Hospital, Liverpool, United Kingdom; ^3^Pädiatrische Rheumatologie, Klinik und Poliklinik für Kinder- und Jugendmedizin, Universitätsklinikum Carl Gustav Carus, TU Dresden, Dresden, Germany; ^4^Zentrum für Pädiatrische Rheumatologie am Klinikum Stuttgart, Stuttgart, Germany

**Keywords:** kawasaki disease, IvIg therapy, vasculitis, inflammatory disorders, fever without infection

## Abstract

Kawasaki disease (KD) is an acute-onset systemic vasculitis of medium-sized vessels that mostly affects infants and toddlers. Globally, it is the most common form of childhood primary vasculitis. Delayed diagnosis and treatment results in coronary artery aneurysms in up to 25% of all affected individuals. Thus, KD is the most common acquired heart disease in developed countries. Here, the current understanding of clinical presentations, pathophysiological concepts, disease-associated complications, and available pharmaceutical treatment is provided and discussed in the context of available literature.

## Background

**Figure 1:**
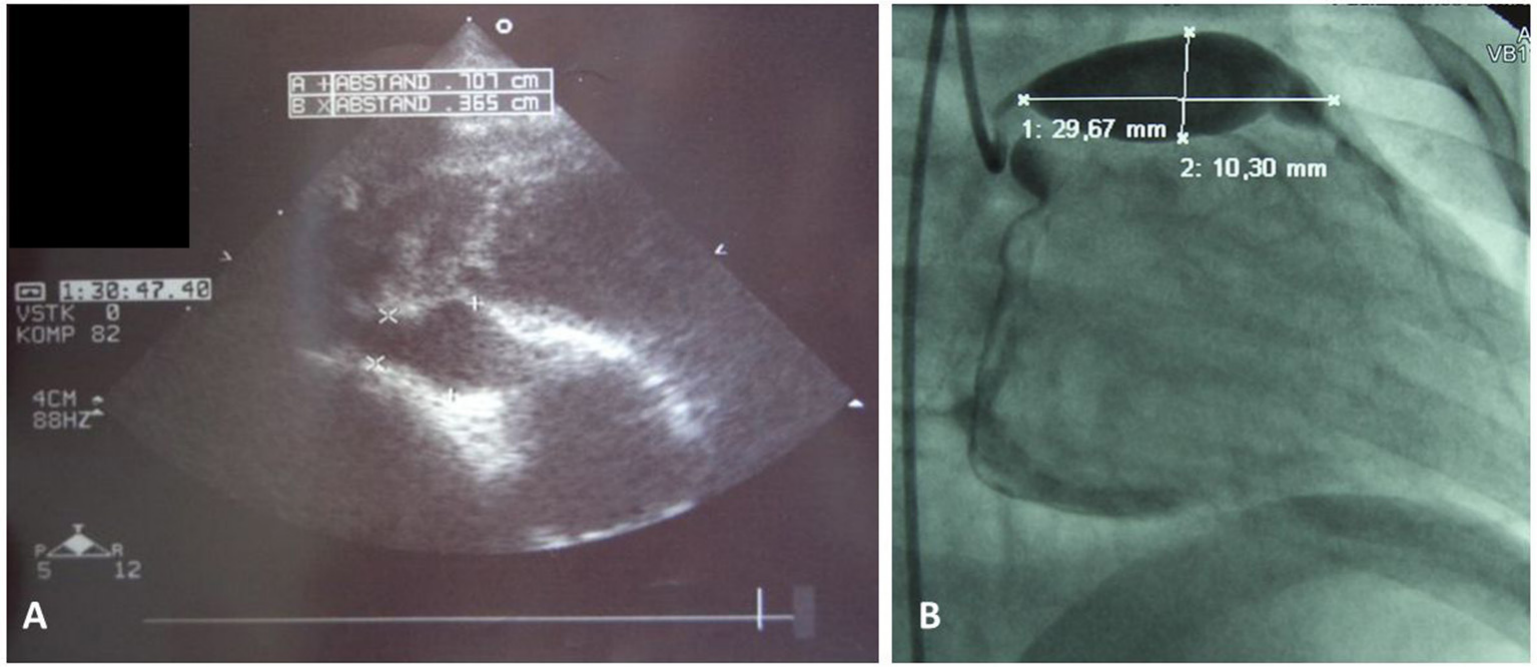
Coronary artery aneurysms. **(A)** Cardial ultrasound of a 3 year old male showing right coronary vessel with aneurysm, and **(B)** classical coronary angiography in a 2 year old male unveiled giant aneurysm of the left coronay vessel.

Kawasaki disease (KD) is a systemic vasculitis mostly affecting medium-sized arteries. Main symptoms include fever, conjunctivitis, skin and mucous membrane affection, and cervical lymphadenopahty. The name KD goes back to the detailed description of 50 children experiencing this form of vasculitis by Tomakisu Kawasaki in 1967 (1). Generally, inflammatory changes to arterial vessels of all body regions can be present, however, coronary arteries are most commonly affected (2). In cases of delayed treatment, missed diagnosis, or in treatment refractory cases, aneurysms can result and cause severe sequelae, including cardiac infarctions (Figure 1, Box 1). Globally, KD is the most common primary childhood vasculitis, in central Europe and North America it is the second most common form (after Henoch Schoenlein Purpura; HSP). To date, KD is considered the most common acquired cardiac condition in childhood in developed countries (3, 4).

Box 1 **Clinical course of an initally undiagnosed patient with KD.**

A 3 year-old Caucasian boy exhibited fevers over 7 days (up to 40°C). In the presence of generalized exanthema and tonsillopharyngitis, the diagnosis *Scarlet fever* was made, and he was treated with aminopenicilline. During the clinical course, reddish swelling of the palms and plants developed. On day 7, the patient was admitted to a small community hospital (with no pediatric rheumatology service). On the day of admission, one brief febrile episode was recorded (38.3°C). On subsequent days, the boy remained afebrile. At admission, reduced general condition, no skin changes, but purulent pharyngitis were recorded. Symmetric conjunctivitis and cervical lymphadenopathy were seen. Bloodwork unveiled elevated CRP levels of 116.6 mg/l and mild thrombocytosis of 464.000/μl (at discharge: 697.000/μl). The patient was diagnosed with tonsillopharyngitis and discharged after several days in good general condition. Cardiac ultrasound was not performed.

Two months after the event, the boy was admitted to the pediatric intensive care unit in cardiac shock. Cardiac ultrasound and subsequent angiography unveiled coronary artery aneurysms and thromboembolic vessel occlusion. Because of persistent cardiac failure, cardiac transplantation was necessary and successfully performed.

## Epidemiology

Though reported globally and not sparing any ethnicities, KD is most common in Japan. Incidence in Japan is approximately 240/100.000 children under 4 years-of-age (5). In North America (USA), KD incidence ranges around 17/100.000 (6). In Caucasian populations incidences range around 9/100.000 (7, 8). Approximately 85% of KD patients are younger than 5 years with an average of approximately 2 years. However, cases in younger and older individuals have been reported (9). Young patients under 12 months have an increased risk for the development of coronary artery aneurysms (up to 60% if untreated). This is particularly concerning, since disease presentation in this young age group is frequently “incomplete” and patients fail to be diagnosed correctly (8, 10).

## Etiology and pathogenesis

The etiology of KD is not known. The presence of familial clusters and increased incidence in Asian populations indicate the presence of a genetic component (5, 11). Associations with genetic variants have been established in various populations (Table 1). Genetic variants in the transforming growth factor (TGF) pathway (*TGF*β*2, TGF*β*R2, SMAD3*) are associated with an increased risk for the development of coronary aneurysms in European KD patients (15, 16). Taken together, it appears that genetic risk for the development of KD and coronary aneurysms may be influenced by variants in several genes that link to immunological pathways. Genetic susceptibility may vary between populations which may explain increased incidences in Asian populations.

**Table 1:** Genetic associations in KD.

**KD Gene association**	**Population**	**Predicted effects**	**References**
*BLK*	Japanese, Taiwanese, Korean	Increased B cell receptor signaling	(12)
*CASP3*	Japanese, Taiwanese, Korean, Chinese, white Americans	Reduced gene expression with effects on cell death	(13)
*CD40*	Japanese, Taiwanese, Korean	Risk allele with increased gene expression, subsequently increased immune activation	(12)
*FCGR2A*	Europeans, Taiwanese, Koreans, Chinese	Reduced binding affinity of low-affinity FCG receptor 2A with effects of immune complex clearance	(14)
*HLA* class II	Japanese, Taiwanese, Korean	Immune activation through antigen presentation	(12)
*IPTKC*	Japanese, Taiwanese, Korean, Chinese, white Americans	Negative regulator of NFAT signaling pathway; risk allele results in icnreased NFAT signaling	(15)

*BLK, Gene encoding for the tyrosine-protein kinase BLK also known as B lymphocyte kinase; CASP3, Caspase 3 gene; CD40, Cluster of differentiation 40 gene; FCGR2A, Gene encoding for the low affinity immunoglobulin gamma Fc region receptor 2A; HLA, human leukocyte antigen (HLA); IPTKC, Gene encoding for the inositol 1,4,5-trisphosphate 3-kinase C*.

Seasonal and regional clusters, and reported associations with wind directions suggest exogenous factors contributing to disease expression in genetically predisposed individuals (17, 18). It has been suggested that infectious agents with low transmission rates or penetrance may cause subclinical disease in many individuals, while causing KD in genetically predisposed children. One line of research suggests the presence of RNA virus infections in KD patients (19, 20). Cytoplasmic inclusion bodies in bronchial epithelia from KD patients contain RNA that could deliver a disease-associated pathogen. However, intensive efforts to characterize failed to deliver an explanation. Furthermore, attempts to identify disease-causing pathogens from patients‘blood or endothelial walls have not been successful (21).

Another previously considered explanation was the presence of a superantigen response to endothelial cells (22). The fact that bacterial strains, particularly *Streptococcus* and *Staphylococcus spp*. produce superantigens supported this hypothesis in light of seasonal clusters in winter. Nevertheless, the only human disease clearly demonstrated to be caused by superantigen exposure to date is toxic shock syndrome (23).

Activation of innate immune cells is an early event in KD and reflected by the increased numbers of neutrophilic granulocytes in the periphery and increased expression and release of pro-inflammatory cytokines IL-1β, IL-6, and TNF-α (19). Over the natural course of KD, effector memory and central memory T cells expand in numbers during the acute phase of KD, while effector memory T cells reduce in numbers during the covalescent phase. This indicated activation of the adaptive immune system during the course of KD (24). Furthermore, IVIG treatment coincides with increased numbers of regulatory T cells, which may be central during the termination of inflammatory responses in KD (25). Whether the emergence of effector T cells in the peripheral blood during the acute phase and the association of cessation of fever after IVIG administration and increased numbers of regulatory T cells during the covalescent phase are indicative of previosuly discussed pathogens or a result of global immune activation in response to initially prevalent innate immune mechanisms remains to be determined.

Taken together, genetic variants predispose to the development of KD. Additional factors appear to be necessary for disease expression. Whether KD is triggered by infectious agents in genetically predisposed individuals or whether it is a genetically complex primary autoinflammmatory disorder currently remains an unanswered question.

## Clinical presentation and diagnosis

**Figure 2:**
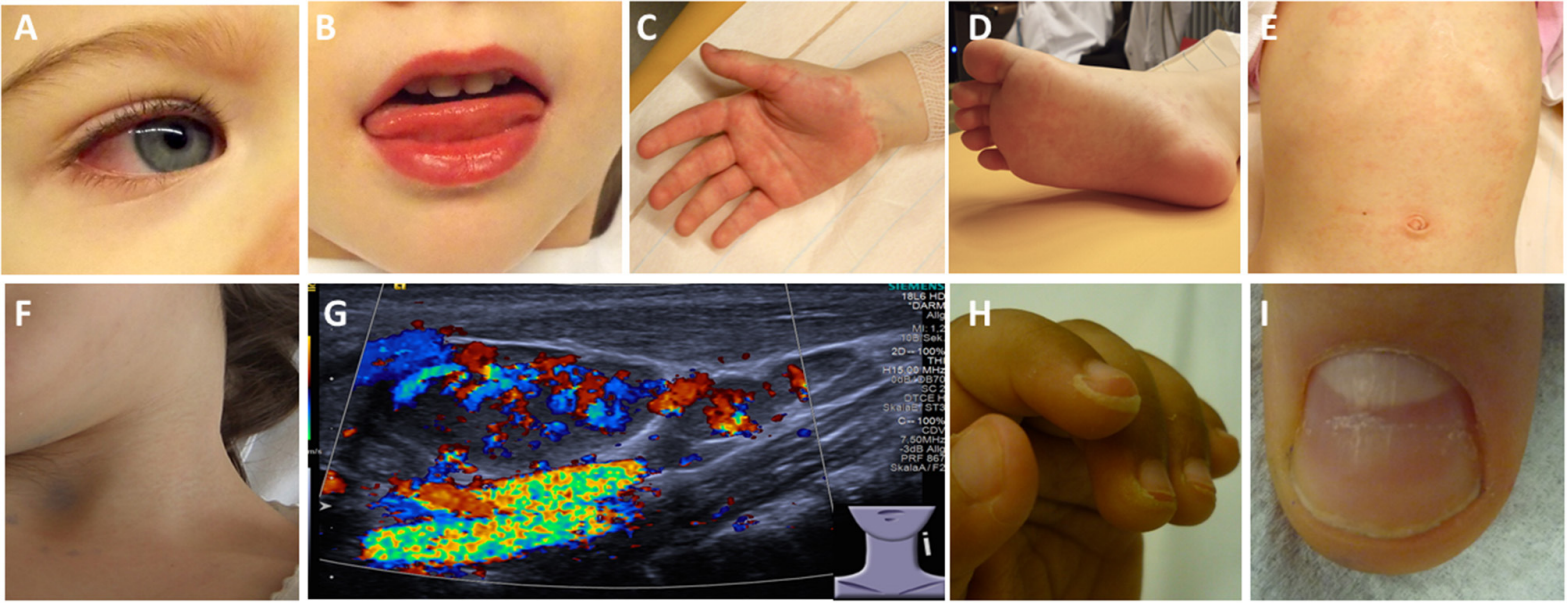
Clinical criteria in KD. **(A)** Bilateral non-purulent conjunctivitis (80–90%), **(B)** changes to oropharyngeal mucous membranes, including injected and/or fissured lips, strawberry tongue (80–90%), **(C)** Palmar and/or **(D)** plantar erythema **(E)** polymorphous exanthema, primarily truncal, not vesicular (>90%), and **(F, G)** cervical lymphadenopathy (>1.5 cm) (50%). **(G)** Ultrasound of enlarged cervical lymph nodes with increased perfusion. **(H)** Periungual desquamation (in covalescent phase) (80%), **(I)** Beau lines.

**Figure 3:**
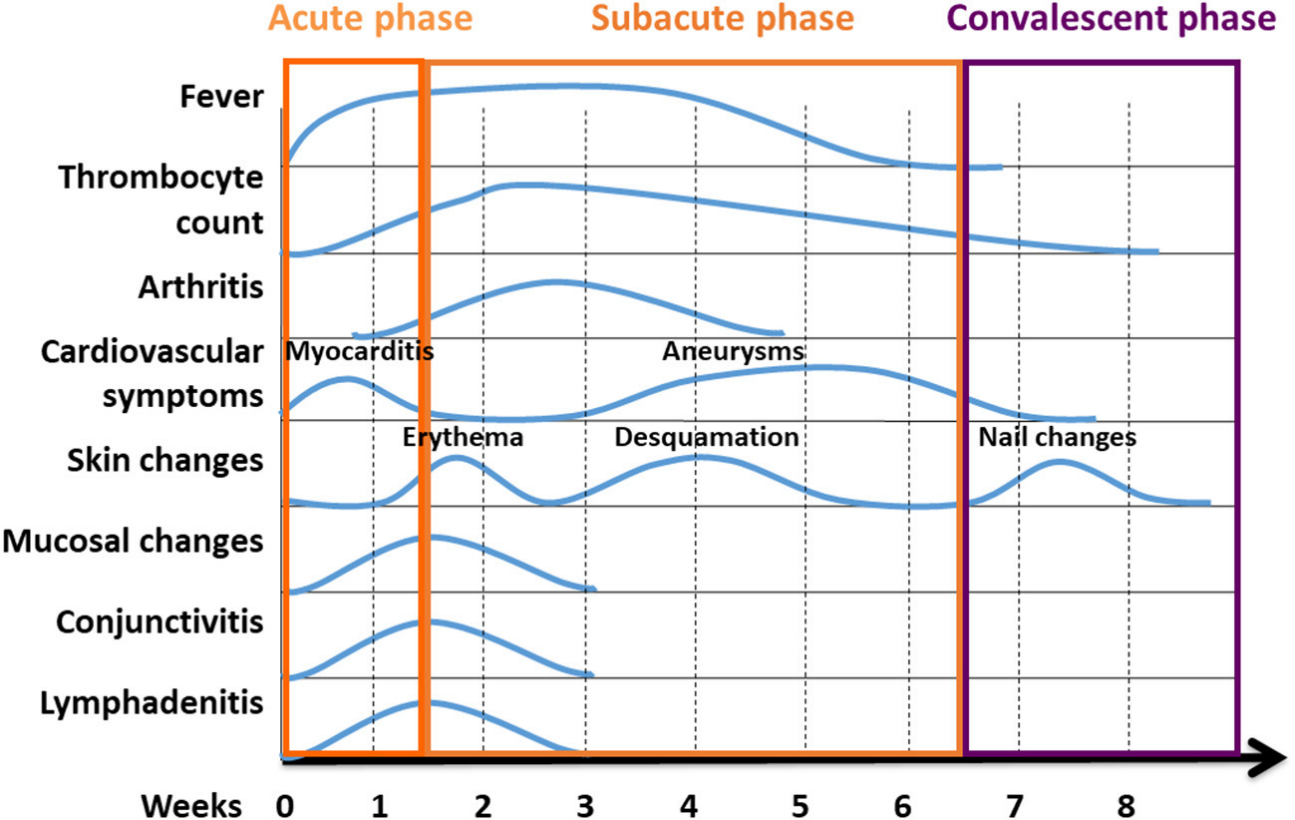
Phases of KD are characterised by variable clinical symptoms. Kawasaki disease reconstitutes a systemic inflammatory disorder with an acute, subacute, and convalescent/recovery phases. Clinical symptoms vary over the course of disease. Classical temporal characteristics are typical of the course of the disease [Figure modified from (23)].

In all cases, KD begins with acute-onset high fever, reduced general condition and frequently reduced cooperativity of children which can complicate physical examination. Further symptoms include generalized polymorphic exanthema (>90%), palmoplantar erythema (80%), symmetric non purulent conjunctivitis (80–90%), usually unilateral cervical lymphadenopathy (>1.5 cm; 50%), and mucosal enanthema with red and/or chapped lips (80–90%) (26) (Figure 2). Additional symptoms include anterior uveitis that can occur in up to 80% of patients (27), and arthritis of small joints (in up to 15%) (28). Later, after several weeks, periungual and/or perianal desquamation, and nail anomalies (Beau lines) can occur (29) (Figure 3). In such cases, the diagnosis can be made based on clinical criteria (Box 2).

Box 2 **Clinical criteria for the diagnosis of “classical” KD (30–33).**

Fever of unknown origin for ≥5 days plus 4 of the following if not explained by another condition. The diagnosis can also be made on day 4 day in the presence of ≥4 principal clinical criteria, particularly when redness and swelling of the hands and feet are present)

- Bilateral Conjunctivitis (80–90%)
- Changes to oropharyngeal mucous membranes, including injected and/or fissured lips, strawberry tongue and enanthema (80–90%)
- Palmar and/or plantar erythema and/or periungual desquamation (in covalescent phase) (80%)
- Polymorphous exanthema, primarily truncal, not vesicular (>90%)
- Cervical lymphadenopathy (at least one lymph node >1.5cm) (50%)

Criteria for the diagnosis of KD are provided in Box 2 and Figure 2.

As also true in other autoimmune/inflammatory conditions, aforementioned definitions do not cover all patients with KD. Up to 36% of patients do not fulfill the diagnostic criteria for KD and can therefore easily be missed. Unfortunately, this group of patients with “incomplete KD” exhibit a particularly high risk for the development of complications, particularly coronary artery aneurysms (34, 35). Particularly young patients with disease-onset in the first 12 months of life delevop these incomplete pictures (36).

**Figure 4:**
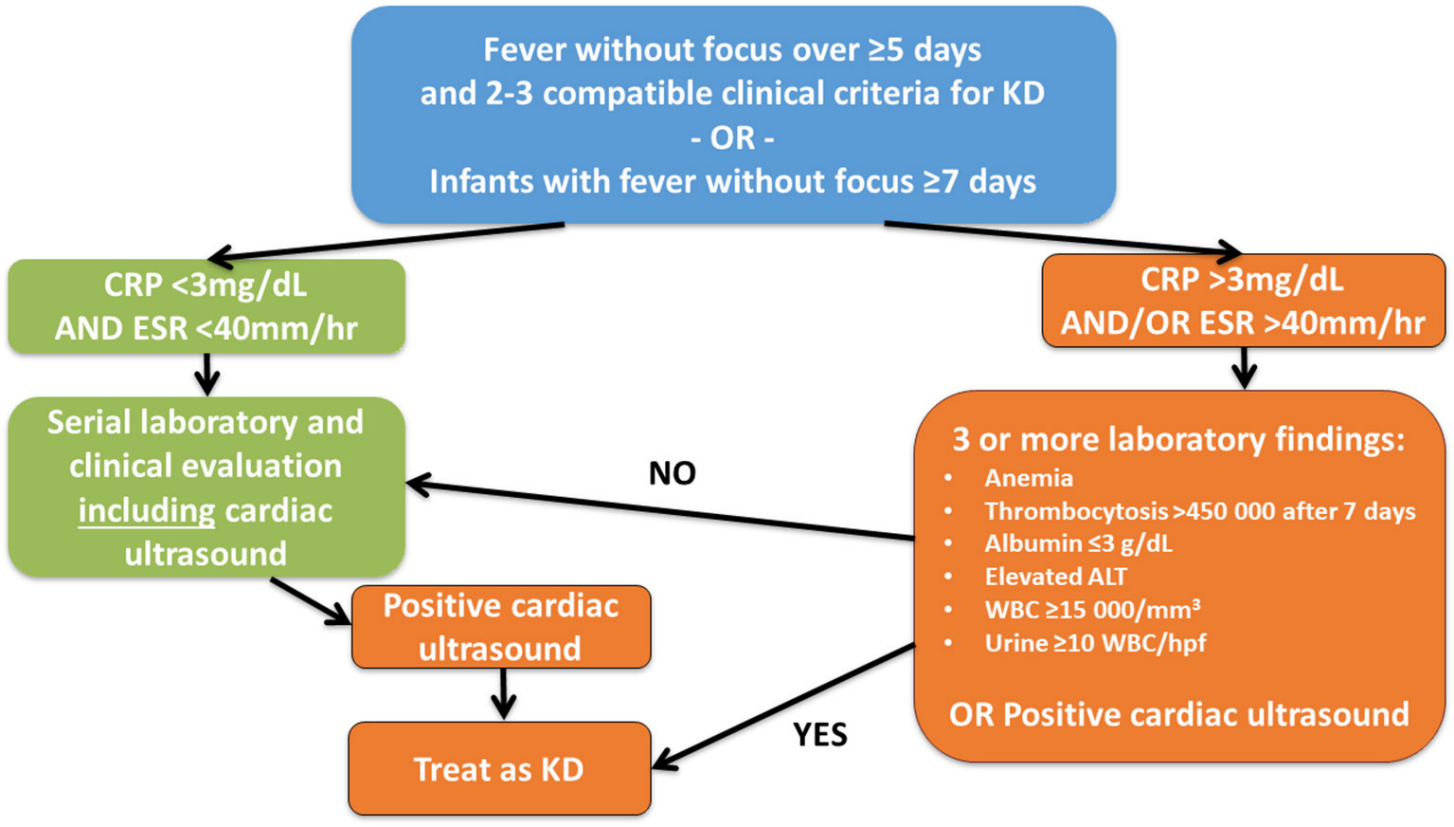
Suggested diagnostic algorithm in suspected incomplete KD. While generally following the suggestions of McCrindle et al. (4), the authors feel that cardiac ultrasound should be performed in all children with fever without focus over ≥5 days and ≥2 clinical criteria for KD or infants with fever without focus ≥7 days independent of CRP levels at least initially.

For patients with suspected incomplete KD, McCrindle et al. (4) suggested diagnostic pathways (Figure 4). Of note, some patients may show atypical clinical findings like exsudative pharyngitis or exsudative conjunctivitis (see Box 1) which make throroughly repetive clinical and laboratory examination mandatory for not missing the diagnosis. For these cases the term “atypical KD” is recommended (37).

**Figure 5:**
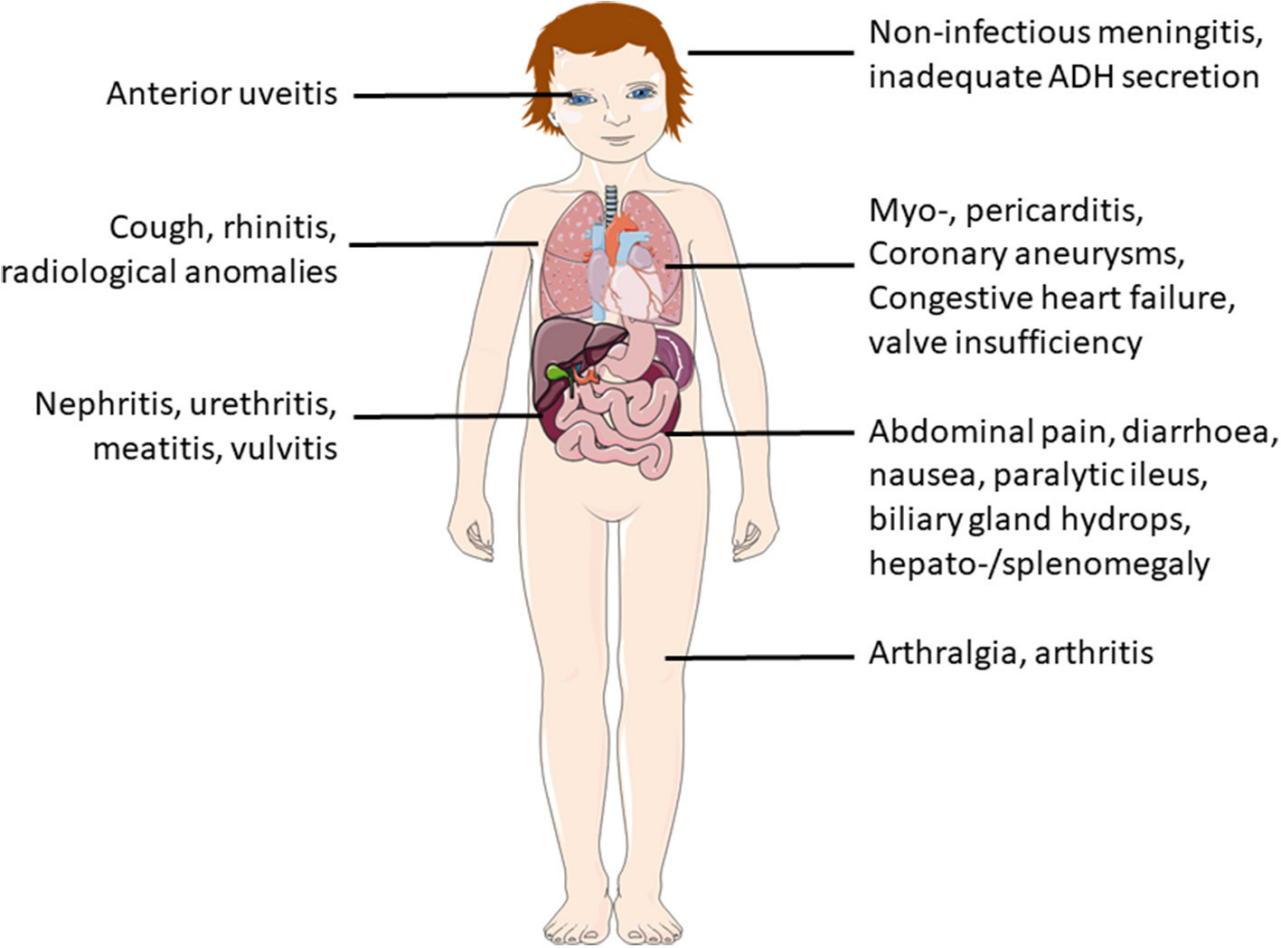
Additional symptoms and possible organ involvement in KD.

Most severe complications usually involve the heart. In the acute phase, a majority of patients exhibit clinically inapparent myocarditis. Arrythmia and cardiac failure, however, are uncommon in this phase (38). Additional organ manifestations include pancreatitis, urethritis, facial palsy and/or macrophage activation syndrome. Ophthalmologic exams should be performed, since anterior uveitis can occurr in up to 80% of KD patients. Non-specific symptoms, such as nausea, diarrhea, abdominal pain (61%), cough and rhinitis (35%) may be present and can delay making the correct diagnosis (28, 37) (Figure 5).

Laboratory investigations and imaging techniques can help to make the correct diagnosis (particularly in incomplete KD) and to exclude important differential diagnoses. Laboratory investigations usually provide evidence for systemic inflammation. Within the first 10 days, almost 80% of KD patients exhibit elevated CRP levels (≥30 mg/l) and ESR (≥40/h). Some patients exhibit elevated liver enzymes (ALT ≥50 U/l), hypoproteinemia and reduced plasma lipid levels, elevated cholestasis parameters (GGT ≥40, hyperbilirubinemia), thrombocytosis (≥450.00/μl), leukocytosis (≥15.000/μl), and/or anemia (Figure 4). In cerebrospinal fluid pleocytosis can be seen in more than 1/3 of KD patients (26, 39). Elevated NT-proBNP (N-terminal pro-brain natriuretuc peptide) levels correlate with increased risk for elevated coronary arteries and may be used as predictive biomarker in the future (40). Serum electrolytes should be tested because inadequate ADH secretion can result in hyponatremia (Figure 5, Box 3) (37).

Box 3 **Controversy about Kobayashi score in non-Asian populations.**

The Kobayashi risk score was tested in a small ethnically mixed population of KD patients the USA. The retrospective analysis delivered high specificity (87%) but low sensitivity (33%). However, only 3 out of 9 patients could be included in the statistical analysis (41). Another small study from the UK delivered specificity of 35%, and sensitivity of 58%. Also this population was small and only included 59 patients (42). Though patient numbers in the two available studies were small and predictive impact is therefore limited, the Kobayashi score failed to identify high risk patients in ethnically mixed populations.

Recently, data from a population-based survey in Germany (ESPED) in the years 2013 and 2014 (301 children at diagnosis, 177 children with follow-up data over 1 year) suggested low prognostic value for the development of persistent coronary aneurysms (after 1 year) of three available scores: Kobayashi, Egami and Sano scores (43). Though overall predictive values were low, scores may be helful to predict courses refractory to IVIG treatment in individual patients.

Cardiac ultrasound is required in suspected KD. Though coronary aneurysms usually develop after several weeks (Figures 1, 3), pericardial effusions or coronary arteries with pronounced vessel walls can be detected early in disease (44, 45). Abdominal sonography can detect intraabdominal effusions and biliary hydrops (37).

## Differential diagnoses

Generally, fever without focus over several days in preschool children should trigger the differential diagnosis KD (Box 2). Diagnosis can be complicated by incomplete or atypical KD. The most common and important differential diagnosis are viral infections. Depending on clinical presentations, infections with Adeno-, Parvo-, Herpes-, and EB-virus need to be considered. In measles, exanthema and enanthema can look similar to KD. In *Scarlet fever*, exanthema, enanthema, cervical lymphadenopathy resemble symptoms of KD. However, purulent tonsillitis and the abscence of conjunctivitis in *Scarlet fever* can help differentiating the two entities (46). Nevertheless caution is needed to not miss atypical cases (Box 1).

Other systemic inflammatory conditions can be differential diagnoses to KD, including systemic juvenile idiopathic arthritis (sJIA). While discriminating between the two entities can be difficult, sometimes the absence of conjunctivitis in sJIA can he helpful.

## Treatment and monitoring

**Figure 6:**
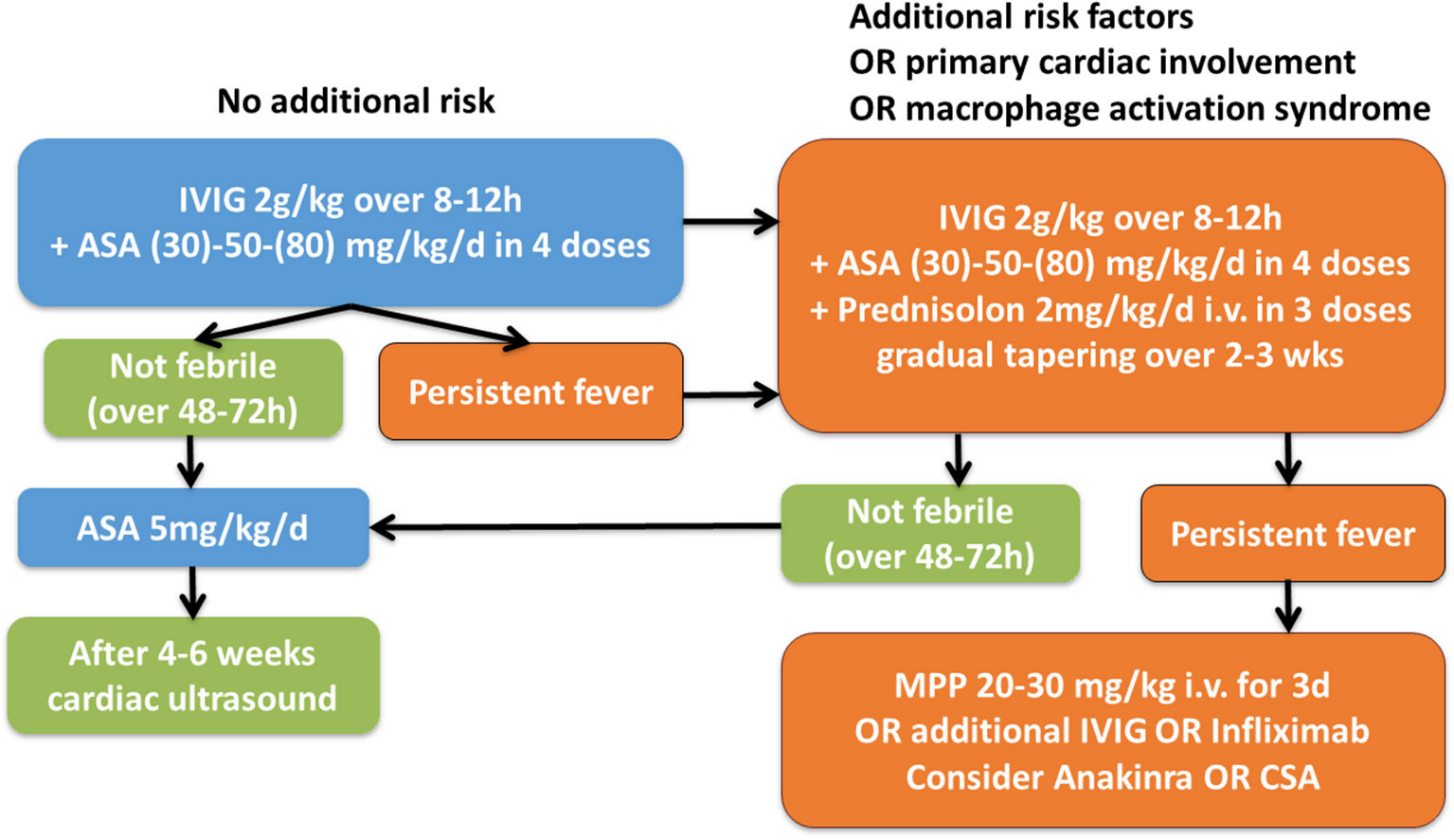
Suggested therapeutic algorithm in KD patients. IVIG, intravenous immunoglobulins; ASA, acetylic acid; MPP, Methylprednisolone i.v. pulse [Modified after (4, 47)].

After making the diagnosis KD, timely treatment with intravenous immunoglobulins is required (IVIG 2g/kg/KG) (Figure 6). It was demonstrated that IVIG treatment, if applied within the first 10 days of fever, reduces the risk for the development of coronary aneurysms from 25% to approximately 5% (48). Furthermore, concommittent treatment with acetylic acid (ASA) (30-)50(-80) mg/kg/day in 4 daily doses is recommended and can be tapered to 3–5 mg/kg/day when patients are afebrile—usually for 48–72 h (49).

Approximately up to 30% of KD patients do not fully respond to these measures (as defined by persisting fever after 48–72 h). Unfortunately, these individuals are at an increased risk for the development of coronary aneurysms (50–53). To identify these individuals, the Kobayashi score was developed. 2 points are given for: Hyponatremia (< 133 mmol/l), elevated GPT (>100/μl), ≤ 4 days of fever before treatment initiation, and severe neutrophilia (>80%); 1 point is given for: young age (<12 months), high CRP (>100 mg/l) and low thrombocyte counts (<300.000/μl). A score of ≥5 correlated with failure to respond to IVIG alone and with increased risk for the development of coronary aneurysms in Japanese children (54). In the same population, oral application of prednisolone until CRP levels normalized significantly reduced the risk for cornary aneurysms (55). These positive effects were supported by a metaanalysis, and none of the included studies suggested significant side effects of oral corticosteroids (56). Of note, positive effects of corticosteroid treatment could not be seen in patients treated with a single i.v. methylprednisolone pulse (57).

For KD patients of Asian descent, various risk scores were developed and showed reasonable sensitivity (77–86%) and specificity (67–86%) for the prediction of IVIG non-response (54, 58, 59). Conversely, two small retrospective studies in mixed ethnic populations in the USA and the UK delivered conflicting results (41, 42) (Box 3).

Based on the observations in two studies in ethnically mixed populations, the value of Kobayashi and other risk assessment scores in non-Asian populations remains unclear. In addition to the aforementioned risk assessment tools, additional associated factors have been reported and include anemia, elevated lactate dehydrogenalse (LDH) levels (>560 U/μl), hyperbilirubinemia >0.9 mg/l), fever for more than 10 days, male gender, and incomplete courses (35, 42, 60, 61). Whether a low threshold for corticosteroid use in non-Asian populations can compensate for reduced sensitivity of risk scores required to be answered. In the authors‘institutions, corticosteroids are currently used at a low threshold in non-Asian KD patients considering the aforementioned risk factors as a final and reliable score for all non-Asian KD patients can currently not be provided. Undoubtedly, all patients with initial cardiac involvement, associated cardiogenic shock, or macrophage activation should receive corticosteroid treatment in addition to “standard treatment” (62).

Several treatment options have been suggested for treatment of refractory cases, which include additional IVIG, corticosteroids, cyclosporine A, and cytokine blocking strategies (63–65). It is worth mentioning that delayed introduction of anti-inflammatory treatment and subsequently prolonged systemic inflammation in KD increases the risk to develop complications (50, 52, 53). Thus, early diagnosis and sufficient treatment are important steps to prevent treatment refractory clinical courses. However, if KD patients fail to respond to standard treatment, either corticosteroids (usually methylprednisolone 20–30 mg/kg intravenously for 3 days with or without subsequent course of tapered oral prednisone) or additional IVIG were reported effective (50, 66, 67). However, beneficial effects were never studied in a randomized clinical trial.

TNF blocking strategies (Infliximab) showed comparable efficacy when compared to a second IVIG course (65). In individual cases, infliximab treatment was associated with regression of coronary aneurysms (68, 69). However, other reports failed to detect beneficial effects in the prevention of coronary aneurysms (70, 71). Furthermore, additional treatment with infiximab on top of IVIG for induction therapy did not reduce the risk for cononary aneurysms in a large randomized prospective study after 5 weeks (72). Taken together, beneficial effects of infliximab on the prevention of cornary aneurysms in KD have not been convincingly documented.

Also, the role of IL-1 blocking strategies has currently not been established, but promise potential. Studies in mice suggest that both IL-1α and IL-1β are centrally involved in the pathophsyiology and development of arterial aneurysms in KD that is preventable by IL-1 blockade (73, 74). Furthermore, beneficial effects of IL-1 blockade have been demonstrated in individual KD cases in humans. A prospective clinical trial investigating effects of treatment with recombinant IL-1 receptor antagonis anakinra in KD patients with early coronary artery involvement (ANAKID trial), however, is not completed yet (75–77). Another interventional clinical trial, aiming to investigate efficacy and safety of canakinumab in pediatric patients KD was withdrawn in 2017.

Calcineurin inhibitors (cyclosporin A, tacrolimus) were tested in small studies (10 and 28 KD patients) and terminated fevers in a subset of otherwise treatment resistant cases. In some cases, calcineurin inhibitor treatment coincided with regression of pre-existing coronary aneurysms (63, 78). However, recent observations in mice, indicating exacerbation of arterial wall aneurysms after calcineurin inhibitor treatment, together with limited experience in human KD raise concerns regharding this treatment option (79). Statin treatment (simvastatin) resulted in significant reduction of CRP levels and coronary dilation in a very small study (80). Sucessful use of cyclophosphamide and plasmapheresis have been reported in individual extremely complicated cases (81, 82).

Acetylic acid (ASA) has been used in the treatment of KD for many years. ASA exhibits anti-inflammatory effects at high doses (50 mg/kg/d), and anti-platelet activity at low doses (3–5 mg/kg/d). Regardless of anti-inflammatory effects, high-dose ASA alone does not reduce the risk of coronary aneurysms development, and should therefore only be given in combination with IVIG and potentially other anti-iflamatory treatment options in KD. Usually, in central Europe and North America, high dose ASA is replaced by low-dose ASA (3–5 mg/kg) after fever eradication. Low-dose ASA should be continued for 6–8 weeks, until coronary ultrasound excludes coronary changes. If coronary aneurysms develop, ASA may be continued indefinitely (4). In the case of large coronary aneurysms, anticoagulant treatment may be considered and is discussed elsewhere (83).

In Figure 6 we provide a suggestion for a therapeutic algorithm based on the available literature, expert opinion, and personal experience of the authors.

Since arterial anuerysms, particularly coronary aneurysms, develop within in the first few weeks after the onset of KD, coronary ultrasound should be performed within and at the end of this period (4–6 weeks after first treatment) (4, 84). Other authors consider altered endothelial function in KD a risk for subsequent coronary disease and recommend “life-style” counceling in regards to atherosclerosis and cardiac ultrasounds every 5 years (85). However, this is currently not part of national or international expert recommendations.

## Prognosis

With timely and adequate treatment (IVIG and ASA), approximately 5% of all KD patients develop arterial aneurysms. Mortality is approximately 0.1% (86). Since delayed diagnosis and treatment initiation are associated with treatment refractory courses and the development of coronary aneurysms, early and sufficient treatment are a key to sucess (50, 52, 53). Generally, cardiovascular risk for KD patients without coronary artery luminal changes is comparable to the general population (87, 88). For all other patients, the severity of luminal anomalies and potentially resulting sequelae (myocardiac infarction, etc.) define the individual risk (4). The risk of relapses in KD is relatively low, and has been reported 2.9% in Japanese children (89).

## Conclusions

Kawasaki disease is a clinically defined systemic vasculitis of mainly medium-sized arteries. Since “incomplete” cases occur, KD should be considered in pre-school children with fever without focus. Delayed or missed diagnosis can result in severe complications, while timely and correct diagnosis and treatment initiation usually garantee a good prognosis. Risk assessment scores are available for Asian populations, however, may miss individual children with increased risk for aneurysm development in other ethnicities. Thus, a low threshold for corticosteroid use may be adviseable in non-Asian populations. Further studies are required and warranted testing effects of cytokine blocking strategies in KD.

## Author contributions

All authors listed have made a substantial, direct and intellectual contribution to the work, and approved it for publication.

### Conflict of interest statement

The authors declare that the research was conducted in the absence of any commercial or financial relationships that could be construed as a potential conflict of interest.
